# Prevalence of Asthma, Allergic Rhinitis, and Atopic Dermatitis and Their Association With Oral Health in Saudi Arabia

**DOI:** 10.7759/cureus.38061

**Published:** 2023-04-24

**Authors:** Seham Alsulami, Amal Aldoboke, Raghad Nooh, Ola Kalifih, Somayya Khan, Osama Marglani

**Affiliations:** 1 College of Medicine, Umm Al-Qura University, Makkah, SAU; 2 Otolaryngology-Head and Neck Surgery, King Faisal Specialist Hospital and Research Centre, Jeddah, SAU; 3 Otolaryngology-Head & Neck Surgery, Umm Al-Qura University, Makkah, SAU

**Keywords:** eczema, oral health, atopic dermatitis, allergic rhinitis, asthma

## Abstract

Background

The word “atopy” is frequently used to describe immunoglobulin E (IgE)-mediated diseases. The prevalence of atopic dermatitis, allergic rhinitis, and asthma is increasing and disconcerting in Saudi Arabia. This study aims to investigate the association between allergic rhinitis, atopic dermatitis, asthma, and oral health among adults in the Makkah region of Saudi Arabia.

Methods

A cross-sectional study of 726 adults using an electronic questionnaire was adopted. The study was carried out from January to December 2022. The questionnaire included demographic data, patients’ diseases in response to inclusion and exclusion criteria, oral health status and symptoms, and dental health-related behaviors.

Results

Most participants were aged from 18 to <40 years (79.1%). More than half of the participants were females (53.6%); 39.7% of participants had poor oral health. Poor health was significantly higher among obese subjects as well as those with lower levels of physical activity, higher perceived levels of stress, those who received a sealant, and those who brushed their teeth for ≤ one time per day. The results showed that the individual symptoms of oral health did not associate significantly with being diagnosed with allergic rhinitis or asthma in the past 12 months. However, atopic dermatitis was independently associated with a chipped or broken tooth (OR = 1.52) and pain in the tongue or inside the cheeks (OR = 3.57).

Conclusion

Poor oral health was significantly associated with atopic dermatitis in Saudi adults. Some systemic diseases are considered chronic diseases, and they are multifactorial; thus, we cannot claim that periodontal pathogens are the definite cause of systemic infections. More studies are necessary to find a definitive association.

## Introduction

The word “atopy” is frequently used to describe immunoglobulin E (IgE)-mediated diseases [1]. Individuals with atopy have an inherited tendency for producing IgE antibodies against common allergens in the environment and have one or more atopic diseases (allergic rhinitis, asthma, and atopic dermatitis) [1]. The prevalence of atopic dermatitis, allergic rhinitis, and asthma is increasing and disconcerting in Saudi Arabia [2].

Allergic diseases can result from interactions between genetic and environmental factors [3,4]. While infectious oral disorders have decreased during the last half-century, allergic diseases have risen significantly [5]. This observation is consistent with the hygiene hypothesis suggesting that fewer chances for infections and microbial exposures have resulted in an increasing prevalence of allergic diseases. Moreover, exposure to oral bacteria, including pathogens associated with gingivitis and periodontitis, may have a protective role in the development of allergic diseases [5]. However, many studies have shown an association between poor oral health and asthma, allergic rhinitis, and atopic dermatitis [6-11].

A significant association between allergic rhinitis and poor oral health has been demonstrated in the Korean adult population [10]. These data also showed that asthma had a significant association and a higher prevalence in the poor oral health group. Moreover, abnormal periodontal status was significantly associated with asthma, and there was also a significant association with allergic rhinitis [10]. A study in the United States found that atopic dermatitis was associated with some dental complaints such as toothache, broken teeth, and gum bleeding [11]. Similarly, studies found that asthmatic patients had more gingivitis and gingival bleeding than non-asthmatic subjects [6,7]. Other studies showed the beneficial effects of oral illnesses on allergic diseases [12-15]. Still others reported a null association between allergic diseases and oral health [16,17]. Therefore, this study aims to investigate the association between allergic rhinitis/atopic dermatitis/asthma with the oral health of the adult population and its prevalence in the Makkah region of Saudi Arabia.

## Materials and methods

This was a cross-sectional, observational study conducted in the Makkah region. Saudi Arabian citizens and residents living in the Makkah region exceeding the age of 18 years were included. Patients with intellectual disabilities and special needs were excluded. Participants were assured of confidentiality and anonymity. The primary objective of this study is to assess the prevalence and association of asthma, allergic rhinitis, and atopic dermatitis with oral health in the Makkah region. The secondary objectives were to define a history of asthma, allergic rhinitis, and atopic dermatitis; to evaluate oral health status and symptoms; and to assess dental health-related behaviors. After obtaining the approval of the Umm Al-Qura University institutional research board, this cross-sectional observational study was performed in the Makkah region in the western part of Saudi Arabia for 12 months in 2022 (from January to December 2022). An electronic survey questionnaire was distributed. We initially received 923 responses on the online platform but excluded nine participants who did not agree to participate, 147 participants who were not residing in the Makkah region, and 41 participants with invalid BMI values. Therefore, data from 726 participants were analyzed further.

Study instrument

The questionnaire included four sections: section one was about demographic data, which comprise gender, age, nationality, marital status, economic level, educational level, city, smoking status, alcohol consumption, BMI, physical activity level, and stress level. BMI was categorized based on the Centers for Disease Control and Prevention (CDC) guidelines into underweight (BMI <18.5 kg/m^2^), healthy weight (BMI 18.5 to <25 kg/m^2^), overweight (BMI 25 to <30 kg/m^2^), and obese (BMI ≥30 kg/m^2^). Section two asked about the patient’s disease (asthma, allergic rhinitis, atopic dermatitis) for inclusion and exclusion criteria, section three had further questions about oral health status and symptoms, and section four was about dental health-related behaviors.

Oral health was assessed using six items based on the World Health Organization (WHO) guidelines. Oral health was classified based on the number of oral symptoms (chipped or broken tooth, toothache, with eating/drinking, throbbing, and sore teeth; sore and bleeding gum, pain in the tongue or inside the cheeks, unpleasant breath) into good (no symptoms), moderate (one oral symptom), and poor (two oral symptoms or more). Regarding dental health-related behaviors participants were asked about whether or not they had received a scaling previous 12 months, whether or not they had received a sealant in the previous 12 months, and the number of times individuals brushed their teeth per day. The frequency of brushing was divided into four groups: ≤ one time a day, two times a day, three times a day, and ≥ four times a day.

Statistical analysis

Statistical analysis was carried out using RStudio (R version 4.1.1; R Core Team (2021). R: A language and environment for statistical computing. R Foundation for Statistical Computing, Vienna, Austria). Categorical variables were expressed as frequencies and percentages. To assess the statistical differences in oral health categories across participants’ characteristics and diagnostic attributes of allergic rhinitis, asthma, and atopic dermatitis, we performed a chi-squared test or a Fisher's exact test. The latter test was used if the expected frequencies were less than five in 50% of cells in a contingency table. We constructed univariate binary logistic regression models to assess the odds of allergic rhinitis, asthma, and atopic dermatitis based on the individual oral symptoms as well as those experiencing at least one oral symptom. The results were expressed as odds ratios (ORs) and 95% confidence intervals (95% CIs). A p-value of < 0.05 indicated statistical significance.

## Results

General characteristics of participants 

The data of 726 participants were investigated in further detail. It showed that more than half of the participants were females (53.6%) and single (60.5%). Most participants were aged 18 to <40 years (79.1%) and were Saudi (89.0%). Most were non-smokers (74.5%) and non-alcohol users (98.8%). Less than half of the participants were overweight or obese (43.0%), and 61.6% of them were performing physical activities for one to 149 minutes per week. In addition, 43.3% of the participants declared that they had moderate to severe stress. More details about the characteristics of participants are listed in Table 1.

**Table 1:** General characteristics of participants SAR: Saudi Riyal; BMI: Body Mass Index

Parameter	Category	Overall, N = 726	Oral Health			
			Good, N = 244	Moderate, N = 194	Poor, N = 288	p-value
Gender	Male	337 (46.4%)	122 (36.2%)	96 (28.5%)	119 (35.3%)	0.076
	Female	389 (53.6%)	122 (31.4%)	98 (25.2%)	169 (43.4%)	
Age	>18 to <40	574 (79.1%)	199 (34.7%)	158 (27.5%)	217 (37.8%)	0.180
	40 to <60	127 (17.5%)	38 (29.9%)	27 (21.3%)	62 (48.8%)	
	60 or more	25 (3.4%)	7 (28.0%)	9 (36.0%)	9 (36.0%)	
Nationality	Saudi	646 (89.0%)	211 (32.7%)	177 (27.4%)	258 (39.9%)	0.279
	Non-Saudi	80 (11.0%)	33 (41.2%)	17 (21.2%)	30 (37.5%)	
Marital status	Single	439 (60.5%)	152 (34.6%)	123 (28.0%)	164 (37.4%)	0.585
	Married	259 (35.7%)	84 (32.4%)	63 (24.3%)	112 (43.2%)	
	Widowed	28 (3.9%)	8 (28.6%)	8 (28.6%)	12 (42.9%)	
City	Makkah	241 (33.2%)	82 (34.0%)	67 (27.8%)	92 (38.2%)	<0.001
	Jeddah	276 (38.0%)	113 (40.9%)	71 (25.7%)	92 (33.3%)	
	Taif	209 (28.8%)	49 (23.4%)	56 (26.8%)	104 (49.8%)	
Economic level (SAR)	< 1,000	154 (21.2%)	53 (34.4%)	47 (30.5%)	54 (35.1%)	0.351
	1,000 to 5,000	221 (30.4%)	72 (32.6%)	61 (27.6%)	88 (39.8%)	
	> 5,000	117 (16.1%)	33 (28.2%)	27 (23.1%)	57 (48.7%)	
	> 10,000	234 (32.2%)	86 (36.8%)	59 (25.2%)	89 (38.0%)	
Smoking status	Nonsmoker	541 (74.5%)	194 (35.9%)	140 (25.9%)	207 (38.3%)	0.222
	Current smoker	135 (18.6%)	35 (25.9%)	42 (31.1%)	58 (43.0%)	
	Past smoker	50 (6.9%)	15 (30.0%)	12 (24.0%)	23 (46.0%)	
Consume alcohol	No	717 (98.8%)	241 (33.6%)	191 (26.6%)	285 (39.7%)	0.916
	Yes	9 (1.2%)	3 (33.3%)	3 (33.3%)	3 (33.3%)	
Educational level	Junior high school or under	20 (2.8%)	3 (15.0%)	3 (15.0%)	14 (70.0%)	0.055
	High school	142 (19.6%)	46 (32.4%)	44 (31.0%)	52 (36.6%)	
	College or over	561 (77.3%)	194 (34.6%)	145 (25.8%)	222 (39.6%)	
	Unknown	3 (0.4%)	1 (33.3%)	2 (66.7%)	0 (0.0%)	
BMI	Underweight	83 (11.4%)	22 (26.5%)	28 (33.7%)	33 (39.8%)	0.001
	Healthy weight	331 (45.6%)	136 (41.1%)	86 (26.0%)	109 (32.9%)	
	Overweight	194 (26.7%)	56 (28.9%)	50 (25.8%)	88 (45.4%)	
	Obese	118 (16.3%)	30 (25.4%)	30 (25.4%)	58 (49.2%)	
Physical activity (per week)	≥150 minutes	188 (25.9%)	78 (41.5%)	48 (25.5%)	62 (33.0%)	0.041
	one to 149 minutes	447 (61.6%)	138 (30.9%)	127 (28.4%)	182 (40.7%)	
	0 minute	91 (12.5%)	28 (30.8%)	19 (20.9%)	44 (48.4%)	
Stress level	No stress	110 (15.2%)	39 (35.5%)	36 (32.7%)	35 (31.8%)	0.005
	Some stress	247 (34.0%)	97 (39.3%)	60 (24.3%)	90 (36.4%)	
	Moderate stress	240 (33.1%)	76 (31.7%)	64 (26.7%)	100 (41.7%)	
	Severe stress	74 (10.2%)	17 (23.0%)	13 (17.6%)	44 (59.5%)	
	Unknown	55 (7.6%)	15 (27.3%)	21 (38.2%)	19 (34.5%)	
Received a sealant in the past 12 months	No	466 (64.2%)	184 (39.5%)	121 (26.0%)	161 (34.5%)	<0.001
	Yes	260 (35.8%)	60 (23.1%)	73 (28.1%)	127 (48.8%)	
Received scaling in the past 12 months	No	474 (65.3%)	156 (32.9%)	126 (26.6%)	192 (40.5%)	0.798
	Yes	252 (34.7%)	88 (34.9%)	68 (27.0%)	96 (38.1%)	
Toothbrushing	≥4 times a day	25 (3.4%)	16 (64.0%)	5 (20.0%)	4 (16.0%)	<0.001
	Three times a day	110 (15.2%)	54 (49.1%)	28 (25.5%)	28 (25.5%)	
	Two times a day	329 (45.3%)	109 (33.1%)	88 (26.7%)	132 (40.1%)	
	≤one time a day	262 (36.1%)	65 (24.8%)	73 (27.9%)	124 (47.3%)	

Factors associated with categories of oral health

Oral health was considered good among 244 participants (33.6%; 95% CI: 30.2 to 37.2), moderate among 194 participants (26.7%; 95% CI: 23.6 to 30.1), and poor among 288 participants (39.7%; 95% CI: 36.1 to 43.3). A significantly higher proportion of Taif city residents had poor oral health (49.8%) than their peers in Makkah (38.2%) and Jeddah (33.3%, p < 0.001). Poor oral health was significantly higher among overweight and obese participants (45.4% and 49.2%, respectively) versus underweight and healthy participants (39.8% and 32.9%, respectively, p = 0.001). The proportions of participants with poor oral health increased consistently with lower levels of physical activity (33.0%, 40.7%, and 48.4% among participants with ≥150 minutes, one to 149 minutes, and no physical activity, respectively, p = 0.041) as well as those with higher perceived levels of stress (31.8% for no stress, 36.4% for some stress, 41.7% for moderate stress, and 59.5% for severe stress, p = 0.005, Table 1).

We found 260 participants (35.8%) received a sealant in the past 12 months, and the proportions of participants who received a sealant increased significantly with oral health based on the number of oral symptoms from good oral health (23.1%), moderate oral health (28.1%), and poor oral health (48.8%, p < 0.001). Moreover, the percentage of participants who brushed their teeth for ≤ one time a day increased significantly from 24.8% among those with good oral health and 27.9% among those with moderate oral health to 47.3% among those with poor oral health (p < 0.001, Table 1).

History of asthma, allergic rhinitis, and atopic dermatitis and their association with the categories of oral health

In the past 12 months, more than a quarter of the participants were diagnosed with allergic rhinitis (25.1%). The diagnosis of asthma was established among 18.5% and atopic dermatitis among 19.0% of them in the past 12 months. The levels of oral health did not differ significantly based on the diagnosis of allergic rhinitis, asthma, and atopic dermatitis in the past 12 months.

Of note, 18.9% of participants self-judged the health of their teeth and gums as poor to very poor. This was consistent with the formal classification of oral health where the proportions of participants with poor oral health increased significantly according to self-perceptions from 12.2% with very good oral health, 33.2% with good oral health, 35.9% with normal oral health, 81.5% with poor oral health, and 100.0% with very poor oral health (Table 2).

**Table 2:** Diagnosis of asthma, allergic rhinitis, and atopic dermatitis and categories of oral health

Parameter	Category	Overall, N = 726	Oral Health			
			Good, N = 244	Moderate, N = 194	Poor, N = 288	p-value
Ever diagnosed with allergic rhinitis	No	515 (70.9%)	175 (34.0%)	144 (28.0%)	196 (38.1%)	0.313
	Yes	211 (29.1%)	69 (32.7%)	50 (23.7%)	92 (43.6%)	
Ever diagnosed with allergic rhinitis in the previous 12 months	No	544 (74.9%)	190 (34.9%)	136 (25.0%)	218 (40.1%)	0.166
	Yes	182 (25.1%)	54 (29.7%)	58 (31.9%)	70 (38.5%)	
Ever diagnosed with asthma	No	613 (84.4%)	205 (33.4%)	165 (26.9%)	243 (39.6%)	0.953
	Yes	113 (15.6%)	39 (34.5%)	29 (25.7%)	45 (39.8%)	
Ever diagnosed with asthma in the previous 12 months	No	592 (81.5%)	204 (34.5%)	154 (26.0%)	234 (39.5%)	0.535
	Yes	134 (18.5%)	40 (29.9%)	40 (29.9%)	54 (40.3%)	
Ever diagnosed with atopic dermatitis	No	583 (80.3%)	202 (34.6%)	160 (27.4%)	221 (37.9%)	0.145
	Yes	143 (19.7%)	42 (29.4%)	34 (23.8%)	67 (46.9%)	
Ever diagnosed with atopic dermatitis in the previous 12 months	No	588 (81.0%)	208 (35.4%)	151 (25.7%)	229 (38.9%)	0.095
	Yes	138 (19.0%)	36 (26.1%)	43 (31.2%)	59 (42.8%)	
The perceived health of the teeth and gums	Very good	131 (18.0%)	80 (61.1%)	35 (26.7%)	16 (12.2%)	<0.001
	Good	196 (27.0%)	82 (41.8%)	49 (25.0%)	65 (33.2%)	
	Normal	262 (36.1%)	78 (29.8%)	90 (34.4%)	94 (35.9%)	
	Poor	130 (17.9%)	4 (3.1%)	20 (15.4%)	106 (81.5%)	
	Very poor	7 (1.0%)	0 (0.0%)	0 (0.0%)	7 (100.0%)	

Odds ratios for three conditions (allergic rhinitis, asthma, and atopic dermatitis) within 12 months according to oral symptoms

The results of the univariate regression analysis showed that the individual symptoms of oral health did not associate significantly with being diagnosed with allergic rhinitis and asthma in the past 12 months. However, an atopic dermatitis diagnosis in the past 12 months was significantly associated with pain in the tongue or inside the cheeks (OR = 2.45, 95% CI: 1.36-4.32, p = 0.002), and having at least one oral symptom of the WHO guidelines (OR = 1.55, 95% CI: 1.03-2.38, p = 0.039). Additionally, having a moderate level of oral health was suggestive of being diagnosed with atopic dermatitis in the past 12 months (OR = 1.65, 95% CI: 1.01-2.70, p = 0.046, Table 3).

**Table 3:** Odds ratios for three conditions (allergic rhinitis, asthma, and atopic dermatitis) within 12 months according to oral symptoms Ref: Reference Category; OR: Odds Ratio; CI: Confidence Interval

Parameter	Category	Allergic rhinitis		Asthma			Atopic dermatitis	
		OR (95% CI)	p-value	OR (95% CI)	p-value	OR (95% CI)		p-value
Chipped or broken tooth	No	Ref		Ref		Ref		
	Yes	1.33 (0.94-1.87)	0.103	1.4 (0.96-2.05)	0.08	1.70 (1.17-2.47)		0.005
Toothache with eating/drinking	No	Ref		Ref		Ref		
	Yes	1.05 (0.73-1.50)	0.771	0.81 (0.53-1.21)	0.306	1.07 (0.72-1.58)		0.729
Throbbing and sore tooth	No	Ref		Ref		Ref		
	Yes	1.12 (0.73-1.70)	0.595	1.35 (0.85-2.12)	0.195	1.22 (0.76-1.92)		0.390
Sore and bleeding gums	No	Ref		Ref		Ref		
	Yes	0.83 (0.56-1.23)	0.364	0.98 (0.63-1.50)	0.942	1.24 (0.81-1.86)		0.315
Pain in the tongue or inside the cheeks	No	Ref		Ref		Ref		
	Yes	0.85 (0.43-1.58)	0.627	0.91 (0.43-1.78)	0.804	2.45 (1.36-4.32)		0.002
Unpleasant breath	No	Ref		Ref		Ref		
	Yes	1.16 (0.78-1.72)	0.453	1.24 (0.80-1.91)	0.325	1.5 (0.98-2.28)		0.057
Having at least one oral symptom	No	Ref		Ref		Ref		
	Yes	1.27 (0.89-1.84)	0.194	1.24 (0.83-1.87)	0.308	1.55 (1.03-2.38)		0.039
Oral health	Good	Ref		Ref		Ref		
	Moderate	1.50 (0.98-2.31)	0.065	1.32 (0.81-2.16)	0.256	1.65 (1.01-2.70)		0.046
	Poor	1.13 (0.75-1.70)	0.555	1.18 (0.75-1.85)	0.478	1.49 (0.95-2.36)		0.087

Since atopic dermatitis diagnosis showed suggestive associations with distinct oral health variables, we next constructed three additional regression models adjusted for selected variables to account for the independent associations. These included Model One (adjusted for gender, age, nationality, marital status, city, economic level, smoking status, alcohol consumption, educational level, and current occupation), Model Two (adjusted for model one in addition to allergic rhinitis and asthma), Model Three (adjusted for Model Two in addition to scaling, sealants, and toothbrushing). The results showed that atopic dermatitis was independently associated with chipped or broken teeth (OR = 1.52, 95% CI: 1.01-2.28, p = 0.043) and pain in the tongue or inside the cheeks (OR = 2.08, 95% CI: 1.09-3.86, p = 0.022) in Model One. Nevertheless, pain in the tongue or inside the cheeks was the sole independent predictor of atopic dermatitis diagnosis in Model Two (OR = 3.36, 95% CI: 1.61-6.91, p = 0.022) and Model Three (OR = 3.57, 95% CI: 1.69-7.46, p < 0.001, Table 4).

**Table 4:** Results of the independent associations between oral health-related variables and an atopic dermatitis diagnosis within 12 months Ref: Reference Category; OR: Odds Ratio; CI: Confidence Interval

Parameter	Category	Crude		Model 1		Model 2		Model 3	
		OR (95% CI)	p-value	OR (95% CI)	p-value	OR (95% CI)	p-value	OR (95% CI)	p-value
Chipped or broken tooth	No	Ref		Ref		Ref		Ref	
	Yes	1.70 (1.17-2.47)	0.005	1.52 (1.01-2.28)	0.043	1.44 (0.87-2.39)	0.151	1.52 (0.90-2.56)	0.116
Toothache with eating/drinking	No	Ref		Ref		Ref		Ref	
	Yes	1.07 (0.72-1.58)	0.729	0.99 (0.64-1.50)	0.949	1.15 (0.68-1.93)	0.604	1.19 (0.70-2.02)	0.514
Throbbing and sore tooth	No	Ref		Ref		Ref		Ref	
	Yes	1.22 (0.76-1.92)	0.390	1.01 (0.60-1.64)	0.982	0.95 (0.51-1.70)	0.860	0.96 (0.51-1.76)	0.885
Sore and bleeding gums	No	Ref		Ref		Ref		Ref	
	Yes	1.24 (0.81-1.86)	0.315	1.20 (0.77-1.85)	0.422	1.45 (0.85-2.47)	0.171	1.53 (0.89-2.63)	0.123
Pain in the tongue or inside the cheeks	No	Ref		Ref		Ref		Ref	
	Yes	2.45 (1.36-4.32)	0.002	2.08 (1.09-3.86)	0.022	3.36 (1.61-6.91)	0.001	3.57 (1.69-7.46)	<0.001
Unpleasant breath	No	Ref		Ref		Ref		Ref	
	Yes	1.5 (0.98-2.28)	0.057	1.50 (0.95-2.36)	0.079	1.48 (0.83-2.60)	0.175	1.61 (0.89-2.90)	0.110
Having at least one oral symptom	No	Ref		Ref		Ref		Ref	
	Yes	1.55 (1.03-2.38)	0.039	1.42 (0.91-2.23)	0.125	1.38 (0.81-2.39)	0.243	1.50 (0.86-2.67)	0.156
Oral health	Good	Ref		Ref		Ref		Ref	
	Moderate	1.65 (1.01-2.70)	0.046	1.61 (0.96-2.71)	0.070	1.46 (0.78-2.73)	0.238	1.56 (0.83-2.97)	0.171
	Poor	1.49 (0.95-2.36)	0.087	1.28 (0.79-2.11)	0.320	1.32 (0.72-2.43)	0.369	1.45 (0.77-2.75)	0.250

## Discussion

The main intention of this study is to investigate the association between allergic rhinitis/atopic dermatitis/asthma with oral health. The results showed that 39.7% of participants had poor oral health. This was higher among obese people, those with lower levels of physical activity, those with higher perceived levels of stress, those who received a sealant in the past 12 months, and people who brushed their teeth ≤one time per day.

An atopic dermatitis diagnosis demonstrated a significant association with various oral health variables. The results after adjustment showed that atopic dermatitis was independently associated with chipped or broken teeth (OR = 1.52) and pain in the tongue or inside the cheeks (OR = 2.08) in Model One. Nevertheless, pain in the tongue or inside the cheeks was the single independent predictor of Model 3 of atopic dermatitis diagnosis (OR = 3.6). A recent study suggested that the oral cavity may have a role in atopic dermatitis [18], and studies have demonstrated that children with atopic dermatitis had worse dental health than children without atopic dermatitis [11,19]. Considering 60% of atopic dermatitis patients had a mutation in the filaggrin gene (FLG), which is expressed in the oral mucosa [20]. Mutations in this gene may also contribute to the etiology of dental caries, which precede chipped and broken teeth. It is believed that a lack of filaggrin in the oral mucosa would increase a person's vulnerability to dryness and infections carried on by germs like Streptococcus mutans, the main cause of dental caries [19,21-23]. A study of Swedish adults showed a positive association between atopic dermatitis and conditions of the oral cavity such as caries, bleeding gums, periodontitis, sensitive teeth, and dry mouth [24].

Over a quarter of the participants had been diagnosed with allergic rhinitis (25.1%) in the past 12 months. Contrary to the findings of Wee et al. [10], our study showed that the individual symptoms of oral health did not correlate significantly with being diagnosed with allergic rhinitis and asthma. Furthermore, a study of Korean adults discovered a significant and inverse relationship between allergic rhinitis and periodontal health (adjusted OR = 0.8). They added that while their findings were consistent with the hygiene hypothesis, it was more applicable for children than adults because the hygiene hypothesis suggests that early-life infections have protective effects against the development of allergies [14].

Most recent evidence showed a clinical association between periodontal disease and several systemic illnesses. Some systemic illnesses are considered chronic diseases. They are multifactorial in nature, and thus they cannot claim that periodontal pathology mechanisms are the definite cause of systemic infections [25].

One strength of this study is that it is the first in Saudi Arabia to study the association between oral health and allergic disease. It also has a large sample size. However, the survey was based on self-reported questionnaires prone to a recall bias, and our study was limited by its cross-sectional design. Therefore, additional research is required to find a definite association between oral health and allergic diseases.

## Conclusions

This study concluded that atopic dermatitis diagnosis demonstrated a significant association with various oral health variables such as chipped or broken teeth and pain in the tongue or inside the cheeks. It also showed that the individual symptoms of oral health did not associate significantly with being diagnosed with allergic rhinitis or asthma. Thus, additional studies are needed to find a definitive association.
